# A Fast-Transient-Response NMOS LDO with Wide Load-Capacitance Range for Cross-Point Memory

**DOI:** 10.3390/s22239367

**Published:** 2022-12-01

**Authors:** Luchang He, Xi Li, Siqiu Xu, Guochang Pan, Chenchen Xie, Houpeng Chen, Zhitang Song

**Affiliations:** 1 School of Microelectronics, University of Science and Technology of China, Hefei 230026, China; 2 State Key Laboratory of Functional Materials for Informatics, Shanghai Institute of Micro-System and Information Technology, Chinese Academy of Sciences, Shanghai 200050, China

**Keywords:** fast transient response, low-dropout regulator, cross-point memory, flipped voltage amplifier, load capacitance

## Abstract

In this paper, a fast-transient-response NMOS low-dropout regulator (LDO) with a wide load-capacitance range was presented to provide a V/2 read bias for cross-point memory. To utilize the large dropout voltage in the V/2 bias scheme, a fast loop consisting of NMOS and flipped voltage amplifier (FVA) topology was adopted with a fast transient response. This design is suitable to provide a V/2 read bias with 3.3 V input voltage and 1.65 V output voltage for different cross-point memories. The FVA-based LDO designed in the 110 nm CMOS process remained stable under a wide range of load capacitances from 0 to 10 nF and equivalent series resistance (ESR) conditions. At the capacitor-less condition, it exhibited a unity-gain bandwidth (UGB) of approximately 400 MHz at full load. For load current changes from 0 to 10 mA within an edge time of 10 ps, the simulated undershoot and settling time were only 144 mV and 50 ns, respectively. The regulator consumed 70 µA quiescent current and achieved a remarkable figure-of-merit (FOM) of 1.01 mV. At the ESR condition of a 1 µF off-chip capacitor, the simulated quiescent current, on-chip capacitor consumption, and current efficiency at full load were 8.5 µA, 2 pF, and 99.992%, respectively. The undershoot voltage was 20 mV with 800 ns settling time for a load step from 0 to 100 mA within the 10 ps edge time.

## 1. Introduction

**Figure 1:**
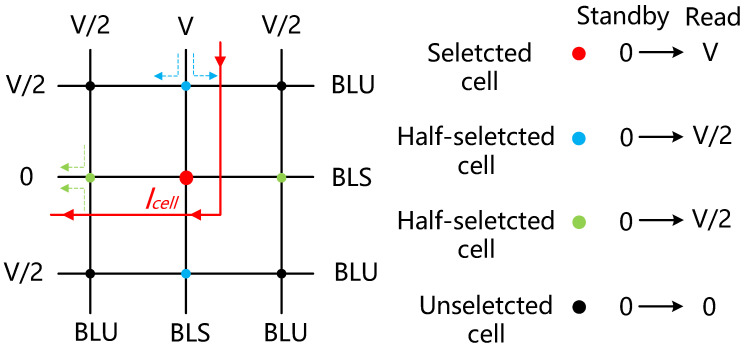
V/2 bias scheme for the read operation.

With the commercialization of Intel’s Optane SSD [1], cross-point memory has attracted great interest for its high density, high capacity, and low latency. In the one-selector-one-resistor (1S1R) configuration, the memory density can reach the smallest cell size of 4 F^2^ (F is the feature size of a technology node) [2]. However, a critical challenge for 1S1R arrays is the large number of sneak paths that reduce the reliability and performance of the system [3]. To address this issue, on the one hand, Ovonic threshold switching (OTS) devices have shown immense potential with their low-temperature process, ultra-low off current, large drive current density, high selectivity (the ratio of on current and off current), and fast operating speed [4]. On the other hand, V/2, V/3, and other bias schemes are proposed to avoid unintentional read and write operations in cross-point arrays [5,6,7,8]. The typical V/2 bias scheme for the read operation is shown in Figure 1. V/2 bias is applied on all the word lines (WLs) and bit lines (BLs) in the standby phase. When the read-enable pulse occurs, the selected BL and selected WL turn to V and the ground, respectively. Meanwhile, the load current of the V/2 bias is transiently changed from no load to heavy load. Therefore, a reliable V/2 bias with a fast transient response is essential for the read operation.

In the V/2 bias scheme, the load current is limited since the sneak path current only exists in half-selected memory cells. In addition, advanced OTS devices with high selectivity can significantly limit the off current [9,10]. The parasitic capacitance of the selector and memory cell is dependent on the material. Moreover, the load capacitance of 1S1R memories is directly related to the size of arrays. As a result, the V/2 read bias needs to remain stable over a wide range of load capacitances.

Compared with the switching converter, a low-dropout regulator (LDO) is more suitable to drive the V/2 bias due to its low noise, small area, and fast transient response. Although novel bias schemes have been proposed for cross-point memory, few circuit-level designs have been presented. To achieve a fast transient response, flipped voltage follower (FVF) topology has been adopted in advanced LDOs [11,12]. A large on-chip output capacitor was adopted to push the output pole to a lower frequency and became the dominant pole. The fully integrated LDOs achieved good transient responses that could respond to less than 10 ns load-transient edges but occupied a large area. A trip-loop LDO based on FVF topology was proposed with only a 0.65 ns response time and 43 mV undershoot when the load changed from 0 to 10 mA within 200 ps [11]. It required an on-chip capacitor of 140 pF, consuming a large chip area. In addition, the power supply rejection (PSR) was only −20 dB at low frequencies. Some LDO systems have been proposed to supply the fast operating bias for nonvolatile memories [13,14]. However, the large on-chip capacitor was not suitable for the cross-point memory with high storage density. An internal dominant pole was designed in a LDO that significantly reduced the chip area [15], but this LDO suffered from instability at zero current load condition and could only maintain system stability within a small range of load capacitance from 0 to 25 pF. Its PSR degraded to −5 dB while reaching 1 MHz. An LDO without an output capacitor and with a direct voltage-spike detection circuit was presented with little overhead [16]. However, the edge time of load switching was 1 µs, which is too long for advanced memory. Recently, NMOS-based LDOs were proposed for low output impedance, better transient responses, and other benefits [17,18,19,20]. To drive the power transistor for low-dropout voltage, charge pumps or extra batteries must be introduced. A charge pump circuit powering the error amplifier would introduce switching noise and consume extra current and area overhead. In the cross-point memory, a read voltage (V_read_) can be set between the threshold voltage of the memory cell in the RESET state (V_tR_) and the SET state (V_tS_). The V/2 bias scheme results in an inevitable voltage difference, so there is no need to minimize the dropout voltage between the input and output voltages during the read operation. For the conventional V/2 bias scheme, the read operation voltages in previous designs ranged from approximately 2.5 V to 4 V [21,22,23]. Therefore, the dropout voltage is high enough to drive the NMOS regulation FET [24]. In summary, exists is a research gap for a fully integrated LDO with a fast transient response and high area efficiency or a non-fully-integrated LDO with high performance to provide a V/2 bias for different sizes of memory arrays.

In this article, a fast-transient-response NMOS LDO based on FVA topology was proposed. The fast FVA loop provides a wide unity-gain bandwidth (UGB), which leads to a short settling time. Meanwhile, the regulator can maintain stability within a wide range of output capacitors from 0 to 10 nF and ESR conditions. This design is suitable to provide V/2 read bias for different sizes of 1S1R arrays.

The rest of this paper is organized as follows. Section 2 describes considerations of the LDO architecture. Section 3 explains the details of the proposed circuit. Section 4 shows the simulation results and a comprehensive comparison. Section 5 concludes the paper.

## 2. LDO Architectural Considerations

### 2.1. NMOS Regulation FET

NMOS utilized in LDOs leads to various benefits. First, compared with a PMOS regulation FET, the gate capacitance (*C*_G_) of the NMOS FET is smaller. Less capacitance helps push the internal poles to higher frequencies. Second, the gate-to-source voltage of the NMOS FET changes directly when the load current varies, leading to a better transient response. Moreover, the source follower structure has a low output impedance so that the output pole can also be pushed to higher frequencies, resulting in a wider loop bandwidth and faster slew rate (SR) when the load changes [19]. Finally, NMOS has a much higher electron mobility, so the NMOS-based LDO shows more area efficiency than the PMOS-based LDO. For a single-supply system, charge pumps are essential in NMOS-based LDO regulators to provide low dropout voltage. Accordingly, NMOS-based LDOs are not commonly used as PMOS-based LDOs due to the introduction of charge pumps that worsen chip area and power consumption. However, the deterioration caused by the charge pump does not exist in this design. V/2 bias scheme provides enough dropout voltage for the NMOS regulation FET to drive the load current at full load condition. In summary, an LDO adopting NMOS can benefit from the small parasitic capacitance, better transient response, low output impedance, wider loop bandwidth, faster slew rate (SR), and increased area efficiency without the cost of a charge pump, which is specific for cross-point memory applications.

### 2.2. Flipped Voltage Amplifier

**Figure 2:**
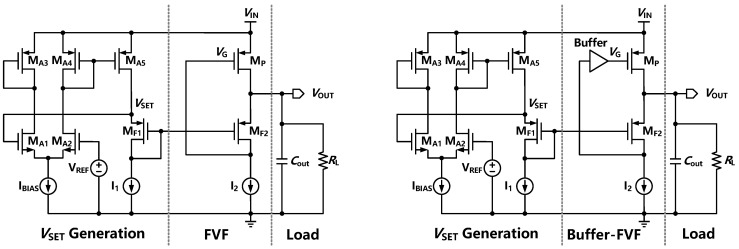
The FVF-based LDO with single-transistor control (left) and inserted buffer (right).

The FVF topology is widely used in on-chip LDOs for fast transient response due to low output impedance, better regulation, and frequency compensation [25]. Two typical FVF-based LDOs are shown in Figure 2 with single-transistor control and inserted buffer. The single transistor control topology is the simplest architecture in FVF-based LDOs that can remain stable under a variety of output capacitors, with a potentially fast transient response and low voltage characteristics. The circuit is mainly composed of *V*_SET_ generation, a flipped voltage follower, and a load module. *V*_SET_ is controlled to be equal to *V*_REF_ by a simple amplifier. Additionally, assume *I*_1_ = *kI*_2_ and (*W*/*L*)_F1_ = *k*(*W*/*L*)_F2_, where *k* is the multiplier of transistors so that the output voltage (*V*_OUT_) can be set the same as *V*_SET_. To improve the stability of the topology and enhance the transient response, a buffer can be introduced to help push the internal poles to high frequencies and set the output pole to be dominant [26].

**Figure 3:**
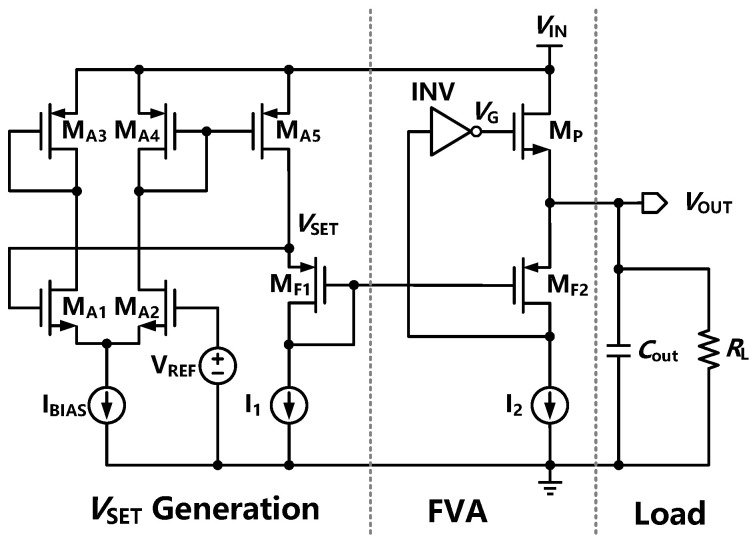
The FVA-based LDO with an inverting amplifier.

Figure 3 shows the architecture of the flipped voltage amplifier-based LDO. First, an NMOS FET is adopted without charge pumps for a lower output impedance and higher unity-gain bandwidth. The DC bias of MF1 and MF2 are the same as those of the FVF-based LDO. The super source follower is replaced by a high-bandwidth inverting amplifier to form a fast negative feedback loop and ensure system stability. In this design, the output capacitor can be a small on-chip capacitor to improve the transient response. The pole at the gate of the NMOS transistor (*p*_gate_) being dominant in the fully integrated FVA-based LDO could provide a stable voltage regulation under a wide range of load capacitance due to the low output impedance. Furthermore, using a large off-chip ESR to push the output pole (*p*_out_) to be the dominant pole, the UGB would be improved compared with FVF-based LDO due to the low output impedance and small parasitic gate capacitance of the NMOS transistor (*C*_gate_). In summary, the FVA-based LDO can remain stable under a wide range of output-capacitor and ESR conditions.

### 2.3. Poles and Stability Considerations

**Figure 4:**
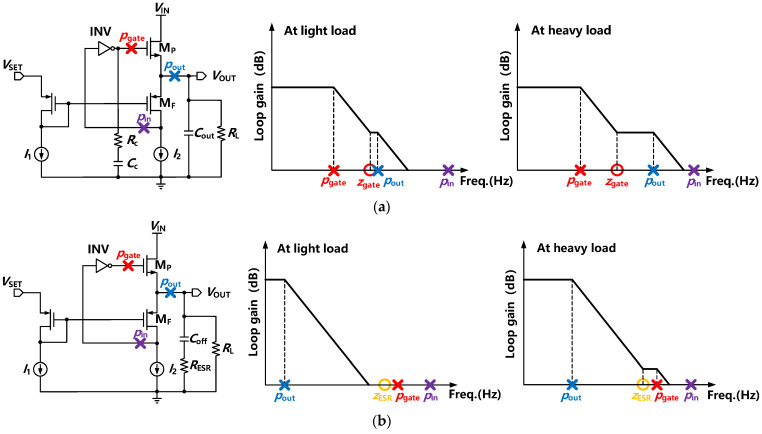
FVF- and FVA-based LDOs with their pole-zero plots. (**a**) FVA LDO with R-C compensation. (**b**) FVA LDO with an off-chip capacitor.

FVF-based LDOs achieve a good transient response within a short edge time compared with traditional resistor-based LDO regulators. However, the stability of the system is dependent on a large on-chip capacitor or ESR capacitance. To avoid significant consumption of area, the dominant pole is designed at the gate of the NMOS within a wide range of load capacitances in the proposed FVA topology. Moreover, the FVA-based LDO adopting the output pole as the dominant pole exhibits superior performance at ESR conditions. The LDO structure and its poles are sketched in Figure 4 under different conditions. Due to the small *C*_G_ and low output impedance of the NMOS FET, two poles *p*_out_ and *p*_gate_ are pushed to high frequencies, as depicted in Figure 4a. Without frequency compensation, the system may face stability problems since *p*_out_ is close to *p*_gate_ at the light load condition. To improve the phase margin at light or no load conditions, small series resistance–capacitance (R-C) compensation was adopted at the gate of the power transistor. This helps to push the *p*_gate_ to a lower frequency and generate a left-half-plane (LHP) zero *z*_gate_. As shown in Figure 4a, *p*_gate_, *p*_out_, and *z*_gate_ can be given by:

(1) $$p_{\mathrm{gate}}=-\frac{1}{\left[C_{C}+C_{\mathrm{gdp}}+C_{\mathrm{gsp}}g_{\mathrm{mbp}}/(g_{\mathrm{mbp}}+g_{\mathrm{mp}})\right]R_{I,C}}$$

(2) $$p_{\mathrm{out}}=-\frac{1}{C_{\mathrm{out}}\left\{\left[1/(1/g_{\mathrm{mp}}+g_{\mathrm{mbp}})\right]//(1/g_{\mathrm{mf}})//R_{L}\right\}}$$

(3) $$z_{\mathrm{gate}}=-\frac{1}{C_{C}R_{C}}$$

where *g*_mp_, *g*_mbp_, and *C*_gsp_ and *C*_gdp_ are the transconductance, body-effect transconductance, and gate-to-source and gate-to-drain capacitances of MP, respectively; *g*_mf_ is the transconductance of MF; *C*_C_, *R*_C_, *R*_L_, *R*_I_, and *C*_out_ are the compensation capacitor and resistance, load impedance, input resistance of the inverting amplifier, and output capacitor, respectively; and *R*_I,C_ is the sum of *R*_I_ and *R*_C_. To maintain the stability of the fast loop of LDO, *z*_gate_ is introduced to compensate for the nondominant pole at light load. Since *p*_out_ is proportional to the load impedance, it depends on 1/(*g*_mbp_ + *g*_mf_) at heavy load conditions. Thus, this nondominant pole is shifted to high frequencies as the load current increases, resulting in a large UGB. Moreover, another nondominant pole *p*_in_ may become nonnegligible due to the wide bandwidth. An on-chip capacitor is added at the output terminal with a small resistance to avoid the effect of the *p*_in_ and enhance the transient response. As the load capacitance increases, the phase margin of the system will gradually deteriorate. Nevertheless, this regulator remains stable with a large output load capacitance.

Figure 4b shows the FVA-based LDO with an off-chip capacitor *C*_off_. In this case, *p*_out_ was designed as the dominant pole due to the large output capacitor. An LHP zero *z*_ESR_ was generated by ESR, which can be expressed as:

(4) $$z_{\mathrm{ESR}}=-\frac{1}{C_{\mathrm{off}}R_{\mathrm{ESR}}}$$

At the light load condition, the dominant pole *p*_out_ is placed at very low frequencies, and the stability is easily satisfied. At heavy and full load conditions, *p*_out_ is shifted to high frequencies as the output resistance decreases, and the nondominant pole *p*_gate_ is lower than the UGB. The LHP zero is utilized to counteract the phase shift of the *p*_gate_. Therefore, loop stability is guaranteed without R-C compensation.

In summary, the FVA-based LDO can maintain stability within a wide range of load capacitance and ESR capacitors. Compared with the FVF-based LDO, at the on-chip output capacitor condition, less capacitor is utilized in the FVA-based LDO for high area efficiency. The UGB of the fast loop is not deteriorated at heavy loads due to the ultra-low output impedance. At ESR conditions, the UGB is significantly improved owing to the lower output impedance and smaller parasitic capacitance. Owing to a wide UGB and direct feedback to the gate-to-source voltage of the NMOS FET, FVA-based LDOs would achieve a fast transient response. In addition, compared with fully integrated LDOs that adopt an internal dominant pole, the FVA-based LDO could remain stable within a wide range of load capacitances and exhibit a better transient response.

## 3. Proposed FVA Low-Dropout Regulator

**Figure 5:**
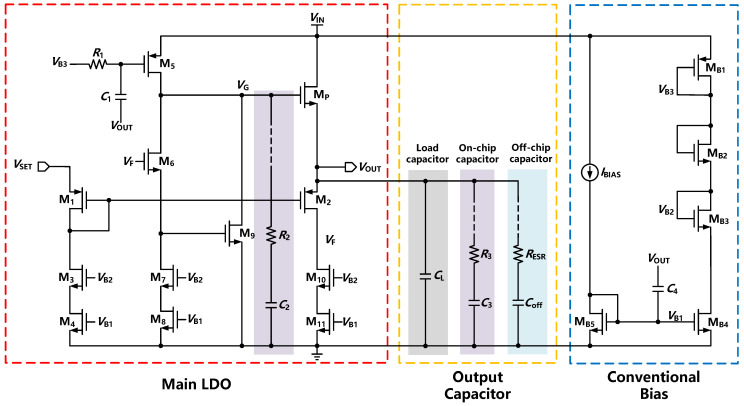
Transistor level schematic of the proposed FVA-based LDO.

Figure 5 shows the transistor level schematic of the proposed flipped voltage amplifier-based LDO. This circuit can be divided into four parts: main LDO, output capacitor, conventional bias, and adaptive bias. Two loops are adopted in the system, including a slow loop to generate the *V*_OUT_ and a fast loop to achieve better dynamics accuracy while load changes. For the slow loop, the *V*_SET_ is generated by a simple error amplifier or a simple LDO circuit that could drive µA-level current. The *W*/*L* ratio of transistors M1 and M2 was set to 1:4, which is the same rate as the bias current generated by the cascode current mirror consisting of M3, M4, M10, and M11. Thus, the source-to-gate voltage of M1 (*V*_SG1_) is equal to the source-to-gate voltage of M2 (*V*_SG2_). Furthermore, *V*_OUT_ is given by:

(5) $$V_{\mathrm{OUT}}=V_{\mathrm{SET}}-V_{\mathrm{SG}1}+V_{\mathrm{SG}2}=V_{\mathrm{SET}}$$

Therefore, *V*_OUT_ can be adjusted according to the setting of *V*_SET_. The slow loop consisting of the above M1, M2, and *V*_SET_ generation circuit could easily satisfy the stability. It provides accurate DC operating points for the circuit and ensures that *V*_OUT_ is equal to *V*_SET_.

Similar to the FVF-based LDO, there is another fast loop to provide fast transient responses when the load current varies. To guarantee the stability of the negative feedback loop, the buffer of the FVF structure is replaced by an inverting amplifier with a wide bandwidth in the FVA topology. The fast loop consists of M2, M6, MP, and current bias. As a result, the NMOS-based LDO has more area efficiency, smaller output resistance, and smaller *C*_G_. The load regulation is less affected by different load currents or capacitances because the power transistor works as a source follower. Moreover, the FVA loop with a high UGB could provide fast transient responses within a short edge time.

The mechanism for the fast loop to respond to current transients is expressed as follows. When the load current changes from light load to heavy load, *V*_OUT_ is lower than the preset voltage, and the drain voltage of M2 drops since the decrease of *V*_SG2_. The gate voltage of M9 drops and the gate voltage of MP increases due to the inverting amplifier. Consequently, the output voltage is forced to increase. In addition, the power transistor can directly provide negative feedback while voltage drops. Meanwhile, when the load current changes from heavy load to light load, *V*_OUT_ is higher than the preset voltage, and the drain voltage of M2 increases as *V*_SG2_ grows. The gate voltage of M9 grows, and the gate voltage of MP is reduced as well. As a result, the output voltage is forced to decrease.

Conventional bias circuits are proposed to provide a reliable current bias for the main LDO module. The conventional bias consists of a simple current mirror and a voltage-spike detection circuit. Additionally, the voltage-spike detection circuit is added at the gate of M5 and the output terminal. The purpose of introducing the voltage-spike detection circuit is to improve the limited SR and transient response without an extra quiescent current. The output voltage is kept constant under a stable load current provided by the conventional bias circuit. When the load current varies, *V*_OUT_ changes and leads to a voltage spike (Δ*V*) rapidly. Therefore, the current is momentarily changed, resulting in an extra dynamic current (Δ*I*) generation. The total and dynamic current can be expressed as follows:

(6) $$I+\Delta I=\frac{1}{2}\mu_{p}C_{\mathrm{ox}}\frac{W}{L}{\left(V_{\mathrm{SG}5}-V_{\mathrm{THP}}+\Delta V\right)}^{2}$$

(7) $$\Delta I\approx \mu_{p}C_{\mathrm{ox}}\frac{W}{L}(V_{\mathrm{SG}5}-V_{\mathrm{THP}}+\frac{\Delta V}{2})\Delta V$$

where *μ_n_*, *C*_ox_, *W*/*L*, and *V*_THP_ are the mobility of electrons, gate oxide capacitance per unit of area, aspect ratio, and threshold voltage of a PMOS device, respectively. Moreover, *C*_1_ and *R*_1_ will introduce a pole-zero doublet at the frequency of 1/*R*_1_*C*_1_, which needs to be set lower than the minimum bandwidth of LDO at different load conditions. The high-pass characteristic of this circuit could increase the low-frequency gain of the fast FVA loop.

Without an off-chip capacitor, the internal pole *p*_gate_ at the gate of the power transistor is designed as the dominant pole in the proposed FVA topology. Meanwhile, *p*_out_ is designed as a nondominant pole due to the super-low output impedance at heavy load. As the load current decreases, the output impedance is increased, and *p*_out_ moves to a lower frequency. Therefore, it is necessary to satisfy stability requirements at light and zero load conditions. The dominant pole *p*_gate_ is high due to the lower *C*_G_ and *p*_out_ getting closer to the *p*_gate_, which leads to insufficient phase margin. To satisfy the stability of the fast FVA loop, R-C compensation is added to introduce an LHP zero *z*_gate_. This zero is designed as lower than *p*_out_ to ensure both UGB extension and loop stability. In addition, the increase in load capacitance would result in lower frequencies of *p*_out_. Nevertheless, the system could remain stable under a wide range of load capacitances due to the small output impedance and R-C compensation. To deal with a faster transient response, the UGB of the FVA loop is designed to be wide. Meanwhile, an on-chip output capacitor is adopted to guarantee a fast transient response and filter out noise from the supply. Additionally, a small resistance is connected to the output capacitor for counteracting the phase shift of the *p*_in_.

**Figure 6:**
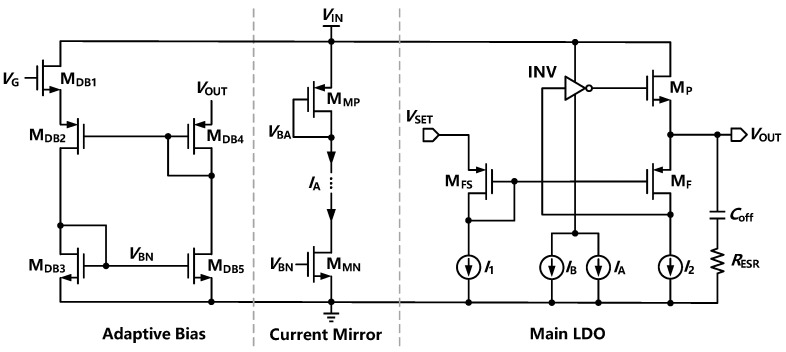
Function of the adaptive bias module.

When a large external capacitor is adopted in FVA-based LDO, *p*_out_ is pushed to a low frequency and becomes the dominant pole. The ESR zero *z*_ESR_ could counteract the phase shift of the *p*_gate,_ and R-C compensation is not required to be adopted. Since a large current would lead to a small output impedance and push *p*_out_ to higher frequencies, an adaptive bias is proposed to improve the stability load regulation within a large load current. Figure 6 expresses the function of the adaptive bias module consisting of current mirrors MDB2 and MDB4. The input current obtained from the sensing load current shows a high accuracy. At a heavy load, adaptive bias is used to improve stability and load regulation. At light load, MDB3 works in the deep subthreshold region, and the adaptive bias circuit consumes ultra-low quiescent current. The generated adaptive current is added to the inverting amplifier for better stability of the FVA loop. In general, the transient response can be effectively enhanced by introducing an off-chip capacitor. This design exhibits a wider UGB and better transient response characteristics than FVF-based LDOs with off-chip capacitors. Meanwhile, it could achieve superior PSR and higher current efficiency. In fact, the introduction of large external capacitors will realize an improved transient response and lower power while sacrificing chip integration.

## 4. Simulation Results

**Figure 7:**
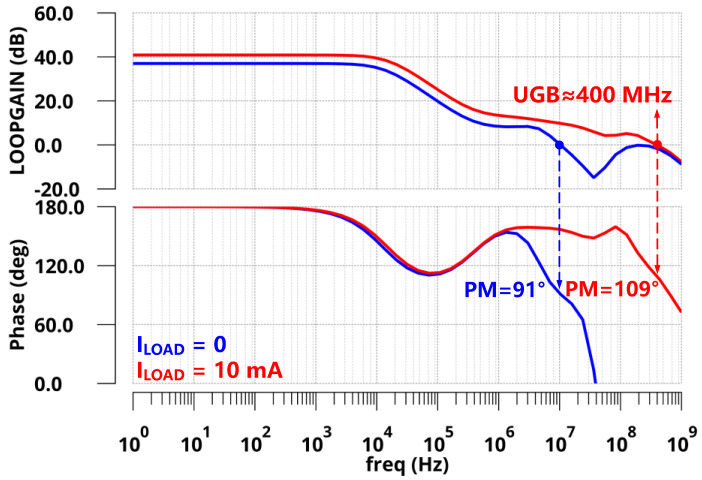
Bode plots of FVA loop responses with zero load capacitance.

**Figure 8:**
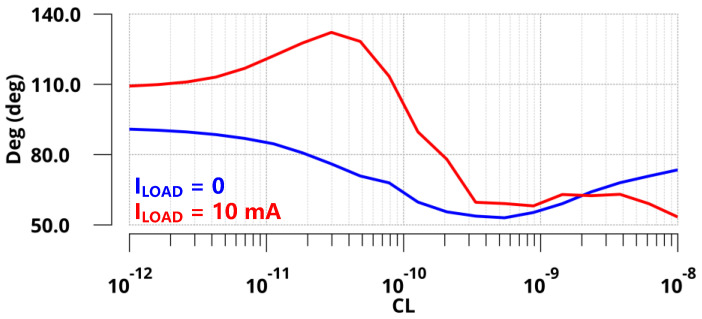
Phase margin of the FVA loop with a range of load capacitances from 0 pF to 10 nF.

The proposed design was implemented in a standard 110 nm CMOS process. At the no off-chip capacitor condition, the FVA-based LDO delivered a maximum load current of 10 mA with a 3.3 V output voltage and 1.65 V output voltage to provide V/2 read bias for cross-point memory. Additionally, the quiescent current was 70 µA. Figure 7 shows the Bode plots of the FVA loops at both zero and full load conditions (*I*_LOAD_ = 0 µA and 10 mA) with zero load capacitance (*C*_L_ = 0 pF). From the simulation, the *z*_gate_ introduced by R-C compensation could counteract the phase shift of the *p*_out_ at both conditions. To address another internal pole *p*_in_ at the heavy condition, a small resistance connected to the output capacitor was adopted to generate an LHP zero at high frequencies. As expected, the phase margins of the FVA loop were 91° and 109° under 0 µA and 10 mA loads, respectively. The UGB of the system reached approximately 400 MHz at full load, leading to fast transient responses. In addition, the pole-zero doublet introduced by the voltage-spike detection circuit in low-middle frequency increases the low-frequency gain. The stability of the system does not deteriorate. To verify the stability of the system under variable load capacitance, Figure 8 presents the phase margin of the proposed LDO with a range of *C*_L_ from 0 pF to 10 nF. The simulation results indicated that the phase margin was larger than 50° at both zero and full load conditions. Therefore, for a wide range of *C*_L_, the proposed LDO is stable at light and heavy load current conditions.

**Figure 9:**
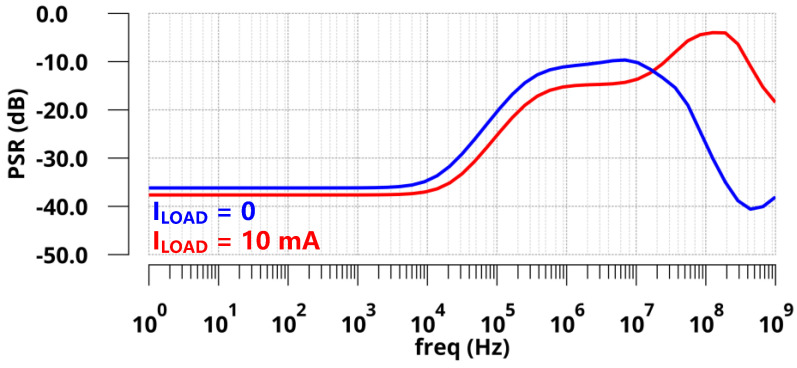
Simulated PSR of the proposed LDO with zero load capacitance.

**Figure 10:**
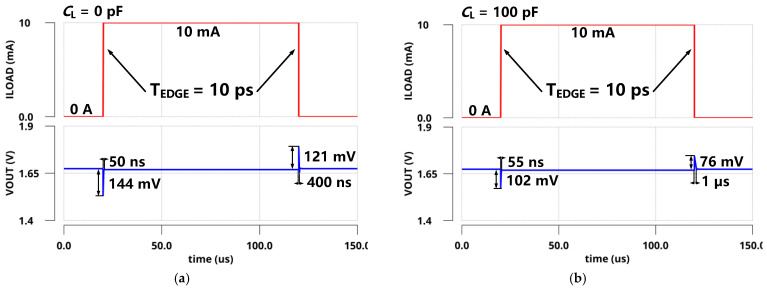
Simulated load transient responses with an on-chip capacitor of 0 and 100 pF within an edge time of 10 ps (**a**) *C*_L_ = 0 pF; (**b**) *C*_L_ = 100 pF.

**Figure 11:**
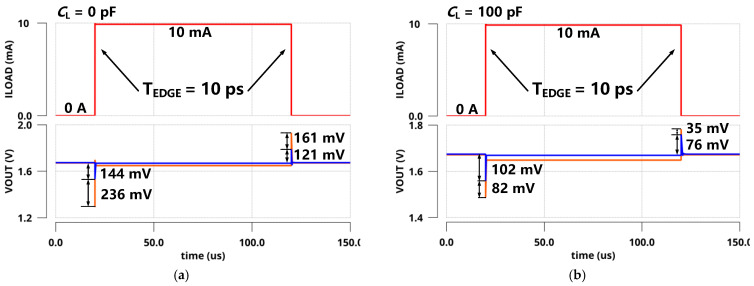
Simulated load transient responses compared with FVF-based LDO within an edge time of 10 ps and the on-chip capacitor of (**a**) *C*_L_ = 0 pF; (**b**) *C*_L_ = 100 pF.

The simulated PSRs of the regulator at zero and full load conditions are presented in Figure 9. With zero *C*_L_, the simulated PSR of the proposed LDO was −17.8 dB at 1 GHz under a 10 mA load and −37.4 dB at 1 GHz under zero load. Figure 10 shows the simulated transient response of *V*_OUT_ with a full current step (*I*_LOAD_ changed from 0 µA to 10 mA) within an edge time of 10 ps. As shown in Figure 10a, the undershoot voltage and settling time were only 144 mV and 50 ns without *C*_L_ due to the fast FVA loop. Additionally, the overshoot voltage and settling time were 121 mV and 400 ns, respectively. As shown in Figure 10b, the simulated undershoot and overshoot voltages were reduced to 102 mV and 76 mV with 100 pF *C*_L_. Furthermore, the simulation results are shown in Figure 11 under the same on-chip capacitor and quiescent current conditions compared with FVF-based LDO. By replacing the PMOS and FVF structure with NMOS and FVA topology, both the undershoot and overshoot were effectively reduced with different load capacitors. As a remark, the proposed LDO exhibits a fast transient response under various load conditions.

**Figure 12:**
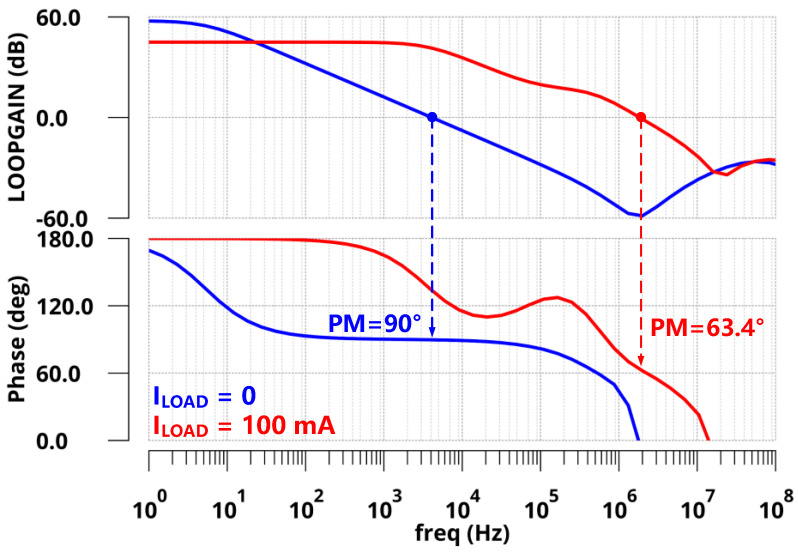
Bode plots of FVA loop responses with a 1 µF off-chip capacitor.

For the ESR case, a large off-chip capacitor of 1 µF with *R*_ESR_ = 100 mΩ was adopted for a large load current and better transient response. Figure 12 shows the Bode plots of the FVA loops at both zero and full load conditions (*I*_LOAD_ = 0 µA and 100 mA). From the simulation, *p*_out_ is pushed to low frequencies and becomes the dominant pole. At the zero load current condition, *z*_ESR_ and *p*_gate_ are locked at frequencies higher than the loop UGB, resulting in a phase margin of 90°, as expected. At the full load current condition, *p*_out_ shifted to higher frequencies owing to the decrease in output impedance. However, *z*_ESR_ could counteract the phase shift of *p*_gate,_ and another internal pole *p*_in_ was higher than UGB. Therefore, the phase margin at full condition is shown as 63.4°.

**Figure 13:**
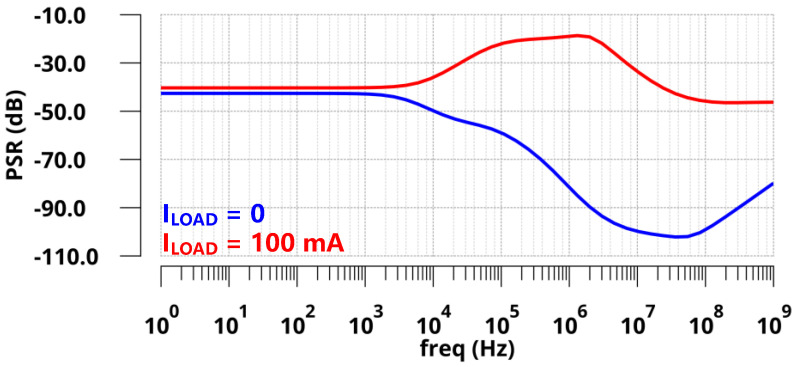
Simulated PSR of the proposed LDO with a 1 µF off-chip capacitor.

**Figure 14:**
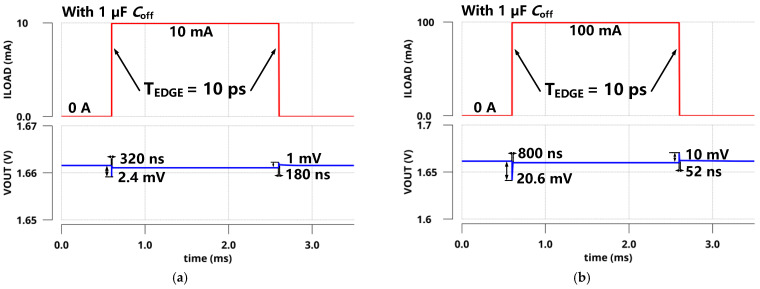
Simulated load transient responses with a 1 µF off-chip capacitor within an edge time of 10 ps for different load steps (**a**) Δ*I*_Load_ = 10 mA; (**b**) Δ*I*_Load_ = 100 mA.

**Figure 15:**
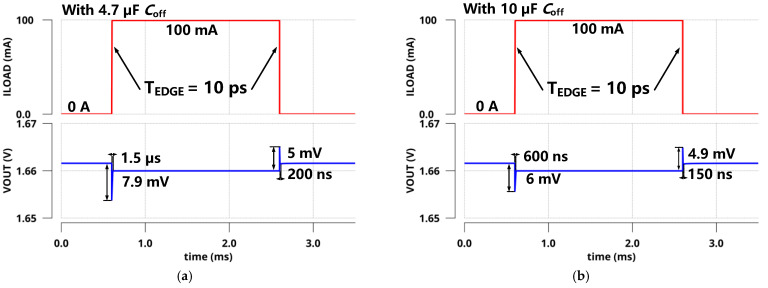
Simulated load transient responses within an edge time of 10 ps and load steps of 100 mA at different ESR conditions (**a**) *C*_off_ = 4.7 µF; (**b**) *C*_off_ = 10 µF.

Figure 13 presents the PSR of the regulator at zero and full load conditions. With the introduction of *C*_off_, PSR was significantly improved, especially at high frequencies. The simulated PSRs of the proposed LDO were −36 dB at 10 kHz and −46.3 dB at 1 GHz under a 100 mA load. Meanwhile, the PSR reached up to −50 dB at 10 kHz and −79.8 dB at 1 GHz under a zero load. Different load steps were applied and are shown in Figure 14 to confirm the transient response characteristics of *V*_OUT_ within an edge time of 10 ps. When the load current changed from 0 µA to a moderate load (10 mA), as shown in Figure 14a, the undershoot and overshoot voltages were 2.4 mV and 1 mV, respectively. In addition, when the load current changed from zero load (0 µA) to a full load (100 mA), as shown in Figure 14b, the undershoot and overshoot voltages were 20.6 mV and 10 mV, respectively. The simulated load transient responses at other ESR conditions are shown in Figure 15. Obviously, this design could guarantee the stability of the system with good performance under the abovementioned conditions. From the simulation results, it is obvious that the transient response of the proposed LDO is significantly improved with the off-chip capacitor.

**Figure 16:**
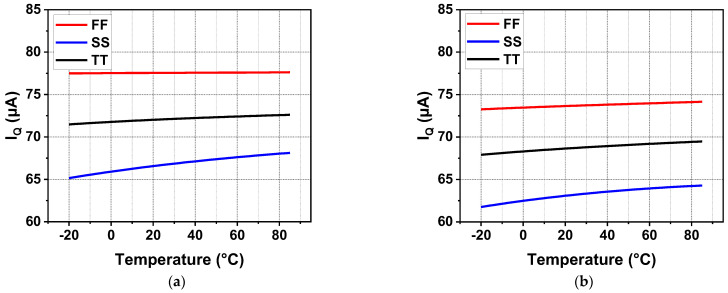
Quiescent current of the proposed LDO without *C*_off_ for different temperatures and process corners at zero and full current load conditions (**a**) at zero load; (**b**) at full load.

**Figure 17:**
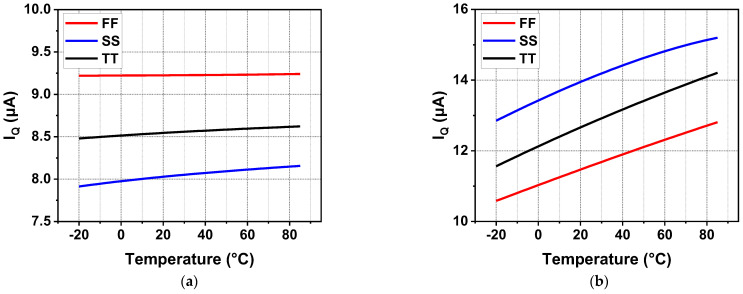
Quiescent current of the proposed LDO with *C*_off_ for different temperatures and process corners at zero and full current load conditions (**a**) at zero load; (**b**) at full load.

Figure 16 shows the quiescent current from −20 °C to 85 °C at different process corners and both zero and full current load conditions. Due to the high gate voltage of MP at the full load condition, the current replicated from the current mirror to the inverting amplifier is slightly reduced. It is obvious that there was a decrease in quiescent current at full load compared to the zero load condition. It reduced the loop gain at low frequencies slightly but did not deteriorate the circuit stability. Similarly, the quiescent current characteristics of the regulator with off-chip capacitors are illustrated in Figure 17. Due to the introduction of *C*_off_, good transient characteristics can be realized without consuming a high current for a high UGB. As a result, the current efficiency is significantly improved. In addition, adaptive bias was adopted to improve the stability and load regulation of the system. Under a light current load, the adaptive bias consumes an ultra-low quiescent current. Meanwhile, under full current load, the increased current consumption is completely acceptable, as shown in Figure 17b.

**Figure 18:**
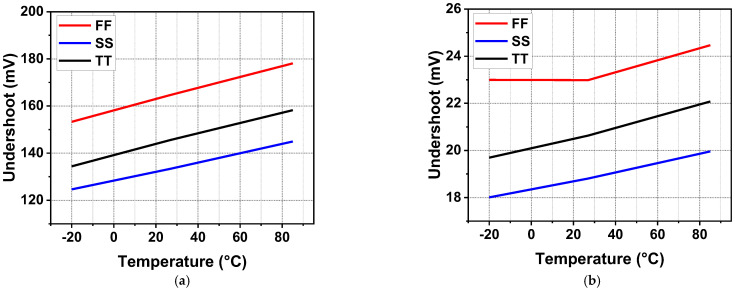
Simulated load-transient undershoots for different temperatures and process corners (**a**) without *C*_off_; (**b**) with *C*_off_.

To further verify the reliability of the transient response for the circuit, the undershoot voltage from −20 °C to 85 °C at different process corners is shown in Figure 18. When the load current changed from zero load to full load, load-transient undershoots could be observed. For the no off-chip capacitor case, the minimum undershoot was 124.6 mV at −20 °C, SS process, and the maximum undershoot was 178 mV at 85 °C, FF process. For a 1.65 V output voltage, the abovementioned undershoot voltage simulation results are acceptable with a quite small settling time. In addition, the load-transient undershoot results of the regulator with off-chip capacitors are illustrated in Figure 18b. The undershoot voltage is significantly reduced, with a minimum undershoot of 18 mV at −20 °C, SS process, and a maximum undershoot of 24.4 mV at 85 °C, FF process.

**Figure 19:**
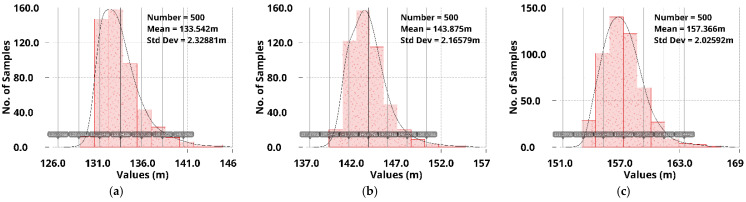
Monte-Carlo simulation results of load-transient undershoots without *C*_off_ for different temperatures (**a**) at −20 °C; (**b**) at 25 °C; and (**c**) at 85 °C.

**Figure 20:**
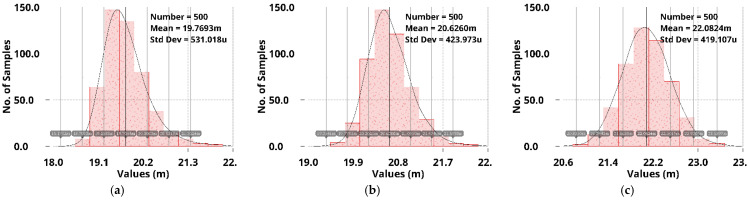
Monte-Carlo simulation results of load-transient undershoots with *C*_off_ for different temperatures (**a**) at −20 °C; (**b**) at 25 °C; and (**c**) at 85 °C.

Simulation results of the 500-run Monte Carlo analysis for mismatch and process variations at −20 °C, 25 °C, and 85 °C are shown in Figure 19 and Figure 20. For the no off-chip capacitor case, the average undershoot µ is between 133 mV and 157 mV, and the standard deviation σ is approximately 2 mV. Moreover, for the off-chip capacitor case, as shown in Figure 20, the average undershoot µ is between 20 mV and 22 mV, and the standard deviation σ is approximately 420 µV.

To evaluate the speed of the transient response, an important parameter response time *T*_R_ is defined in refs. [15,27]. For a fair evaluation, *C*_L_ is replaced by the total capacitance adopted by the LDO *C*_Total_, including the load capacitance and compensation capacitor. In the case that *T*_EDGE_ is much smaller than *T*_R_, the current drawn from *C*_L_ can be seen as a constant. It can be expressed as:

(8) $$T_{R}=\frac{C_{\mathrm{Total}}\Delta V_{\mathrm{OUT}}}{\Delta I_{\mathrm{OUT}}}$$

Another estimation of *T*_R_ is needed for fast LDOs in the case that *T*_R_ < *T*_EDGE_. It reads:

(9) $$T_{R}=\sqrt{\frac{2C_{\mathrm{Total}}\Delta V_{\mathrm{OUT}}T_{\mathrm{EDGE}}}{\Delta I_{\mathrm{OUT}}}}$$

where *T*_EDGE_ is the load-transient edge time. In addition, the figure of merit (FOM) defined in [17] is used as a baseline for LDOs, which is as follows:

(10) $$\mathrm{FOM}=K\frac{\Delta V_{\mathrm{OUT}}(I_{Q}+I_{LMIN})}{\Delta I_{\mathrm{OUT}}}$$

where *K*, *I*_Q_, and *I*_LMIN_ are the ratio of the measured *T*_EDGE_ to the minimum *T*_EDGE_, average value of quiescent current, and the minimum load current, respectively. Table 1 summarizes the comparison between the proposed FVA-based LDO and other state-of-the-art LDOs. For the no off-chip capacitor case, the *T*_R_ of the design is not the smallest compared with other regulators. However, neither the transient characteristics nor the allowable range of the load capacitance meet the requirements of V/2 bias in cross-point memory [15]. For the regulator in [18], the parameter is obtained under the condition that the *I*_L_ changes from 1 mA (not zero load) to the full load current. In addition, the proposed FVA-based LDO without *C*_off_ achieves the smallest FOM of 1.01 mV among previous works and can maintain stability over a wide range of load capacitances. For the off-chip capacitor condition, the transient response characteristics of the FVA-based LDO are also well improved. The smallest *T*_R_ and FOM are achieved compared with other LDOs with *C*_off_.

## 5. Conclusions

A fast-transient-response NMOS LDO with a wide load-capacitance range for cross-point memory was proposed. The fully integrated FVA-based LDO achieved a small undershoot voltage and a fast settling time within an edge time of 10 ps and a small FOM of 1.01 mV. For different selectors and memory devices, it could provide stable V/2 bias in the read operation within the load capacitance of 10 nF. This is the first attempt to propose an LDO for the V/2 bias scheme of cross-point memory. The limited on-chip output capacitor makes it suitable for SoC applications compared with FVF-based LDOs. Moreover, the proposed FVA-based LDO with a 1 µF off-chip capacitor shows a significantly improved performance, indicating that it is suitable for low-integration applications. In summary, the presented design achieved fast-transient-response characteristics and can remain stable with different load capacitances.

## Figures and Tables

**Table 1:** Performance summary and comparison.

Publications	TPE 2018 [15]	TPE 2021 [12]	TPE 2016 [18]	JSSC 2018 [20]	TPE 2020 [28]	TPE 2022 [29]	This Work	
Technology	0.13 µm	65 nm	65 nm	0.25 µm	0.18 µm	0.18 µm	**0.11** **µm**	
Regulation FET	PMOS	PMOS	NMOS	NMOS	PMOS	PMOS	**NMOS**	
*V*_IN_ (V)	1–1.4	1.2	2.4–2.6	1.5–3.3	1.4–1.8	1.8–2.2	**3.3**	
*V*_OUT_ (V)	0.8	1	1	1.0–3.0	1.2	1.6	**1.65**	
*C*_L_	On-chip			Off-chip			**On-chip**	**Off-chip**
	0–25 pF	300 pF	30 pF	1–47 µF	4.7 µF	1 µF	**0–1000 pF**	**1 µF**
*I*_LMAX_ (mA)	25	20	30	150	150	200	**10**	**100**
*I*_LMIN_ (µA)	120	5	0	0	100	0	**0**	**0**
*I*_Q_ (µA)	112	27–82	10	1.24–100	13.5	48	**70**	**8.5–12.5**
*C*_ON-CHIP_ (pF)	5.73	303	39	18.25	N/A	N/A	**48**	**2**
PSR (dB@Hz)	−5 @100 M	−16 @1 G	−12.8 @1 G	<−22 @20 k	−30 @10 k	−45 @10 k	**−17.8** **@1 G**	**−36** **@10 k**
*T*_SETTLE_ (ns)	<190	20	500 *	900	2000	N/A	**50**	**800**
Δ*V*_OUT_ (mV)	322	59	195 *	160	20	76	**144**	**20**
*T*_EDGE_ (ns)	0.3	0.8	0.2	10	500	100	**0.01**	
*K* ratio	30	80	20	1000	50,000	10,000	**1**	
Charge pump	No	No	Yes	Yes	No	No	**No**	
*T*_R_ (ns)	0.21	0.9	0.253 *	1066.7	627	380	**0.69**	**200**
FOM (mV)	52.9	13.9	1.3 *	54	756.8	182.4	**1.01**	**0.002**
Results	Meas.	Meas.	Meas.	Meas.	Meas.	Meas.	**Sim.**	

* *I*_L_ changes from 1 mA (not zero load) to 30 mA (full load).

## Data Availability

Not applicable.
